# “Applying Intersectionality in designing and implementing health interventions: a scoping review”

**DOI:** 10.1186/s12889-021-11449-6

**Published:** 2021-07-16

**Authors:** Elham Ghasemi, Reza Majdzadeh, Fatemeh Rajabi, AbouAli Vedadhir, Reza Negarandeh, Ensiyeh Jamshidi, Amirhossein Takian, Zahra Faraji

**Affiliations:** 1 Community-Based Participatory Research Center, Tehran University of Medical Sciences, Tehran, Iran; 2 Knowledge Utilization Research Center, Tehran University of Medical Sciences, Tehran, Iran; 3 School of Public Health, Tehran University of Medical Sciences, Tehran, Iran; 4 Center for Academic and Health Policy, Tehran University of Medical Sciences, No.12, East Nosrat St., Tehran, Iran; 5 Department of Anthropology, Faculty of Social Sciences, University of Tehran, Tehran, Iran; 6 Founding Member, Center of Excellence in Health Sociology (CEHS), University of Tabriz, Tabriz, Iran; 7 Nursing and Midwifery Care Research Center, School of Nursing and Midwifery, Tehran University of Medical Sciences, Tehran, Iran; 8 Department of Health Management, Policy and Economics, School of Public Health, Tehran University of Medical Sciences (TUMS), Tehran, Iran; 9 Health Equity Research Center (HERC), Tehran University of Medical Sciences (TUMS), Tehran, Iran; 10 Department of Global Health and Public Policy, School of Public Health, Tehran University of Medical Sciences (TUMS), Tehran, Iran; 11 Central library, Iran University of Medical Sciences (IUMS), Tehran, Iran

**Keywords:** Intersectionality, Health inequality, Health policy, Health intervention, Scoping review

## Abstract

**Background:**

Given the potential of intersectionality to identify the causes of inequalities, there is a growing tendency toward applying it in the field of health. Nevertheless, the extent of the application of intersectionality in designing and implementing health interventions is unclear. Therefore, this study aimed to determine the extent to which previous studies have applied intersectionality and its principles in designing and implementing health interventions.

**Methods:**

The title and abstract of the articles which were published in different databases e.g. PubMed, Web of Science, Proquest, Embase, Scopus, Cochrane, and PsychInfo were screened. Those articles that met the screening criteria were reviewed in full text. The data about the application of principles of intersectionality, according to the stages heuristic model (problem identification, design & implementation, and evaluation), were extracted through a 38-item researcher-made checklist.

**Results:**

Initially, 2677 articles were found through reviewing the target databases. After removing the duplicated ones and screening the titles and abstracts of 1601 studies, 107 articles were selected to be reviewed in detail and 4 articles could meet the criteria. The most frequently considered intersectionality principles were “intersecting categories” and “power”, particularly at the stages of ‘problem identification’ as well as ‘design & implementation’. The results showed that “multilevel analysis” principle received less attention; most of the studies conducted the interventions at the micro level and did not aim at bringing about change at structural levels. There was a lack of clarity regarding the attention to some of the main items of principles such as “reflexivity” as well as “social justice and equity". These principles might have been implemented in the selected articles; however, the authors have not explicitly discussed them in their studies.

**Conclusions:**

Given the small number of included studies, there is still insufficient evidence within empirical studies to show the implication of intersectionality in designing and conducting health interventions. To operationalize the intersectionality, there is a need to address the principles at various stages of health policies and interventions. To this end, designing and availability of user-friendly tools may help researchers and health policymakers appropriately apply the intersectionality.

**Supplementary Information:**

The online version contains supplementary material available at 10.1186/s12889-021-11449-6.

## Background

Tackling health inequalities is regarded as one of the most critical missions of health systems globally, among high, middle, and low-income countries [1, 2]. The inequalities have significant effects on the health status, and their economic and social costs are considerable [3]. The impacts of health inequalities have been already approved on the occurrence of the diseases, morbidity and mortality, life expectancy, as well as access to services [4–7]. In order to respond to this global issue, “intersectionality” has been addressed as a theoretical innovation for the analysis of power structures and procedures that might create and maintain inequalities. The origin of intersectionality dates back to the feminist studies on black women and the criticisms toward racial prejudices; it was coined by Kimberle Crenshaw [8–10]. From the viewpoint of intersectionality, human beings are shaped by the interaction of multiple interlocking locations (such as race/ethnicity, gender, social class, age, migration status, & etc.), and these interactions occur within a context of interconnected systems and power structures. Moreover, it can lead to the experience of privileges and oppressions [10]. In other words, it might better reflect the dynamic nature of the individuals’ experiences, inequities, and interactions with the social context [8]. It takes into account the experiences of marginalized populations which can be helpful in the development of cost-effective interventions and policies in health promotion programs [11]. Intersectionality is based on the principles which are likely to challenge and transform the power relations, thus mandates health systems toward being responsible for health inequities [8]. These central tenets include: Human lives cannot be reduced to single characteristics; Human experiences cannot be accurately understood by prioritizing any one single factor or constellation of factors; Social categories/locations such as race’/ethnicity, gender, class, sexuality and ability are socially constructed, fluid, and flexible; Social locations are inseparable and are shaped by interacting and mutually constituting social processes and structures, which, in turn, are shaped by power and are influenced by time and place; and the promotion of social justice and equity is paramount [12].

Based on the literature review, health researchers have utilized this approach in conceptual and empirical studies as well as policy analysis. Intersectionality-based Policy Analysis Framework is regarded as one of the main efforts to apply intersectionality so far. This framework has been introduced as an instrument for policy analysis which is aimed at evaluating different impacts of policy on individuals and groups, as well as their experiences of health inequalities. It helps health researchers and policymakers understand different implications of the policies and promote social justice within a complex and diverse population [12]. Moreover, some review studies have already discussed the reflection of intersectionality in the field of health; for instance, Bowleg investigated the challenges and benefits of intersectionality for public health theory, research, and policy [11]. She described intersectionality as a “critical, unifying, and long overdue theoretical framework for which public health has been waiting”. Through a descriptive-analytical narrative review study, Couto et al. aimed to search for the studies in the fields of public and collective health within different databases and discussed the methodological and theoretical characteristics of intersectionality [13]. The authors concluded that, given the growing health inequalities across the world along with the potential of intersectionality to support empirical studies and formulate health policies committed to social justice, there is a need for the researchers to move towards the incorporation of intersectionality. The evaluated articles in this study consisted of essays with a theoretical or methodological basis, while the empirical studies were excluded. Consequently, the application of intersectionality was not reviewed in the articles with an empirical nature. Larson et al. also reviewed the literature on intersectionality applications in the field of public health; they further introduced 10 of the best resources with an emphasis on low- and middle-income countries (LMICs) [8]. This study provided useful information about the empirical/conceptual nature of the studies, the intersections across different stratifiers, as well as the health topics in the LMICs and HICs. However, it did not provide any clarifications on how intersectionality has been applied in such studies. Moreover, to access the relevant studies in public health, the researchers only looked up the articles through the PubMed database; and therefore, the comprehensiveness of the review was limited.

Overall, given the potential of intersectionality to identify the causes of health inequalities and improve health justice, its application is on the rise in the field of health. Although there is ample theoretical evidence on the notion of intersectionality, it must be sufficiently utilized in practice as well. Moreover, according to the evidence, intersectionality has been widely used in the analysis of inequalities; however, the extent of the application of intersectionality in designing and implementing the interventions and programs is still unclear. Scoping reviews examine the evidence in order to determine the extent and range of a field [14]. In addition to summarizing the available evidence, these types of studies can be used to identify research gaps in the relevant topics [14–16]. The present study was conducted in the form of a scoping review in order to determine the extent to which previous studies have applied intersectionality and its principles in designing and implementing health interventions and programs.

## Methods

Arksey and O’Malley’s methodological framework [14] was used in the present study in order to guide our scoping review and respond to the question of “What is the extent of the application of intersectionality in designing and implementing health interventions and programs?”. Accordingly, different stages of the scoping review were taken into account as follows:

### Identifying relevant studies

#### Search strategy

To identify the relevant studies without any time or language restrictions, in January 2018, the researchers reviewed the existing documents which were published in the following databases: PubMed, Web of Science, Proquest, Embase, Scopus, Cochrane, and PsychInfo. The search strategies were employed through the use of Medical Subject Heading (MeSH) terms using the keywords of “Intersectionality” AND “Intervention” OR “Health program” (Additional file 1). The reference lists of the selected studies were also reviewed.

### Selecting the studies

The studies were then selected by two reviewers, independently, based on the following inclusion criteria: (1) to be a primary research, (2) to conduct a health intervention or program, (3) to clearly state the application of “*intersectionality”* in designing and implementing the intervention or program, (4) to focus on health outcomes (e.g. morbidity, mortality, risk factors, health behaviors, access to health services, & quality of health services), and (5) to address at least two social identity variables (e.g. age, gender, ethnicity/race, social class, migration status, & etc.).

At first, the titles and abstracts of the selected studies were screened. Those studies that met the inclusion criteria were then reviewed in full text. Finally, the relevant studies were included in the review process. It is also noteworthy that any disagreements between the two reviewers would be resolved through discussion, and it would be arbitrated by a third investigator if necessary until they reach unanimity.

### Charting data

The data extraction form was designed to obtain the general and methodological data. In addition, a researcher-made checklist was used to assess the application of intersectionality in each study (Table 1). The checklist was developed based on the key principles and guiding questions of “intersectionality-based policy analysis framework” introduced by Hankivsky et al. [12]. Furthermore, as a result of reviewing the related literature about the application of intersectionality in health and social sciences, the researchers extracted other items in accordance with the seven key principles of intersectionality, i.e., “*intersecting categories”, “multilevel analysis”, “power”, “reflexivity”, “time & space”, “diverse knowledge”, and “social justice & equity”*. The questions in the proposed checklist were arranged according to the stages heuristic model. The stages heuristic model is one of the most well-known and the primary policy analysis frameworks introduced by Laswell. The framework has maintained its validity and prevalence in public policy analysis and has also been used by many policy analysts, academicians, and independent researchers around the world. In this model, the process of producing public policies is divided into different stages such as identification of the problem, designing and implementation, as well as evaluation [17, 18]. Each of the questions of the proposed checklist was discussed and the necessary modifications were made based on multiple sessions held by the research team. Then, upon the consensus, the 38-item checklist of “application of intersectionality approach in health interventions and programs” was finalized. The possible responses to the questions in the checklist were considered as *‘yes’*, *‘no’*, and *‘unclear’*. The *‘unclear’* option would be chosen if the studies did not clearly outline whether the items had been observed or not. Two reviewers were assigned to complete the data extraction form for each of the studies included in the review process. Besides, in case of disagreement, the research team would seek consensus.

**Table 1:** Checklist for application of Intersectionality in health interventions and programs

	Intersectionality Principles	items	yes	No	Unclear
**Problem identification**	**Intersecting Categories**	1-Has the combination of different social factors, such as age, gender, race/ethnicity, class, migration, been addressed in identifying the causes of the problem? And is not focused solely on a single variable, apart from others?			
		2-Have target groups been selected based on considering to differences, variations and similarities between relevant groups? As well, based on that, have target groups been identified as the most vulnerable group?			
		3-In identification of the most vulnerable groups, have the differences and similarities of the subgroups in terms of social factors, been considered?			
	**Multilevel analysis**	4-Have the various factors at the individual, interpersonal, organizational, and governance levels been addressed in the process of problem identification?			
		5-Have the intersections of social factors across micro, meso and macro level been considered? For example, among immigrants as a marginalized group, how the interactions at the individual level (age, gender, race, class,...) link to social institutions and broader structures and processes of power such as migration policies?			
	**Power**	6-Have the most advantaged and the least advantaged groups been identified within representation of problem?			
		7-Have stakeholders such as affected populations been participated in problem identification?			
		8-Have the structures of power such as policies and laws been addressed to be responsible to the framing the health problem?			
	**Reflexivity**	9-Do the planning committee/research team look critically at their values, experiences, beliefs and assumptions, about the health problem?			
	**Time & Space**	10-Has the process of problem framing over time (historically) or across different places (geographically) and changes of privileges and disadvantages, including intersecting identities and the processes that determine their value over time and place, been considered?			
	**Diverse Knowledge**	11-Has the perspective of people who are typically marginalized been used in the process of problem identification?			
		12-Has the knowledge generated from several recourses including qualitative or quantitative research; empirical or interpretive data; and Indigenous knowledge?			
	**Social Justice & Equity**	13-Do current interventions/programs focus on health promotion of vulnerable groups?			
		14-Are existent interventions/programs being considered in terms of being successful in reducing inequality or, conversely, creating inequality? (For example, are there supportive policies or empowerment programs for vulnerable groups?)			
**Design & implementation**	**Intersecting Categories**	15-Has the intervention/program been selected based on identifying problem using intersectional perspective?			
		16-Is the target group representative of the experiences of diverse groups of people for whom the issue under study is relevant?			
	**Multilevel Analysis**	17-Have the researchers/health planners considered the transformation across multiple levels (individual and interpersonal, family, Neighborhood, city)?			
	**Power**	18-Have various stakeholders, in particular affected population, been engaged in health program design and implementation?			
		19-Has the intervention/program been framed within the current cultural, political, economic, societal context? And has it reflected the needs of affected populations?			
		20-Does the intervention/program focus on vulnerable groups?			
		21-Does the intervention/program lead to a change of power relations? (For example, the participation of target groups in decision making and/or policy making)			
		22-Is it clear that who are responsible to ensure the implementation of the intervention/program? In other words, are there mechanisms for accountability (organizational commitment, etc.)?			
		23-Can the intervention/program find a practical position in line with government policy priorities such as budget allocations, ministerial priorities, etc.)?			
	**Reflexivity**	24-Do the researchers/health planners have reflexive practice? In other word, Do they have critical thinking about their values, experiences, beliefs, assumptions, and current actions and decisions?			
	**Time & Space**	25-Is the intervention/program flexible in terms of time and place conditions?			
	**Diverse Knowledge**	26-Have the target group’s knowledge been used in process of health program design and implementation?			
		27-Has the intervention/program been selected based on diverse evidence (academic sources, gray literature, policy reports,…)?			
	**Social Justice & Equity**	28-Has intervention/program been designed and implemented to reduce inequalities?			
		29-Is there assurance that the intervention/program does not lead to produce further inequities for some populations?			
**Evaluation**	**Intersecting Categories**	30-Have intersectional factors been measured in the evaluation process?			
	**Multilevel Analysis**	31-Have the effects of the intervention/program at individual and interpersonal levels, family, neighborhood, and city, been evaluated?			
	**Power**	32-Have affected groups been engaged in the evaluation process?			
		33-Has the intervention/program enhanced the inclusiveness?			
	**Reflexivity**	34-Do the researchers/planners have reflexivity about the values, experiences, beliefs, assumptions, and current actions and decisions related to measuring the effectiveness?			
	**Diverse Knowledge**	35-Has stakeholder perspectives, in particular target groups, about whether the intervention or program has been effective or not, been considered?			
		36-Has the intervention/program been evaluated based on diverse evidence (academic sources, gray literature, policy reports,…)?			
	**Social Justice & Equity**	37-Is the measure of success in intervention/program determined on the basis of reducing inequalities?			
		38-Has intervention/program led to a reduction in inequality?			

### Summarizing and reporting the results

This stage includes the descriptive summary and the synthesis of the study characteristics based on data extraction. A matrix was drawn to summarize the application of the intersectionality principles. To synthesize the application of intersectionality in all the included studies, the total number and the percentage of the responses to each question were outlined and presented in the matrix. The PRISMA Extension for Scoping Reviews - PRISMA-ScR was followed in reporting the findings of our scoping review.

## Results

**Fig. 1:**
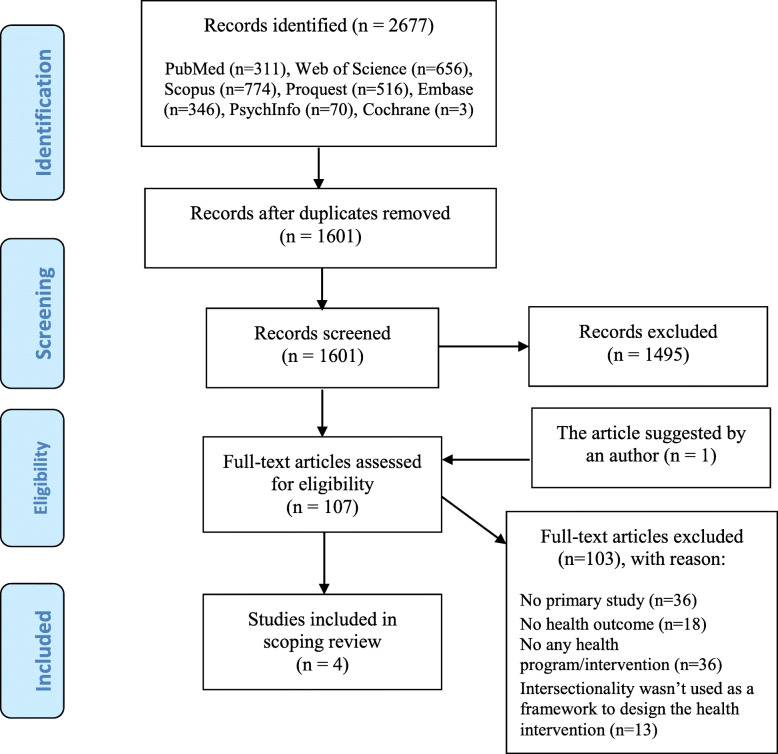
PRISMA flow diagram for the study selection process

Figure 1 illustrates the flowchart for the different stages of study selection based on PRISMA. As a result, 2677 studies were found through reviewing the target databases. After removing the duplicated articles (1076 cases), the titles and abstracts of the remaining 1601 studies were examined during the initial screening stage. Finally, 106 articles were selected for full text assessment according to the inclusion criteria. At this stage, another study was introduced by one of the authors of a related article via email, which was examined as well. However, after assessment of the full text, it was excluded because it did not comply with the inclusion criteria. Eventually, 103 out of the 107 articles were excluded, and only 4 articles were selected for scoping review in the present study.

Table 2 shows that focusing on psychosocial or sexual health outcomes can lead to educational or supportive interventions in the fields of mental [19, 20, 22] or sexual health [21, 22]. In terms of the type of the studies, two interventions were designed as RCT [21, 22] and one study used the pre-test/post-test design [20].

**Table 2:** Summary of the studies included in the present scoping review

Author (year)	Title	Country	Design	Social identities/conditions	Sample size	Sample Characteristics	Intervention/Service	Outcomes	Key findings
David et al. [19]	Safety, Trust, and Treatment: Mental Health Service Delivery for Women Who Are Homeless	USA	Non interventional	-Gender (Women) -Homelessness -Substance use or co-morbid substance use and mental illness	Over 300	Race/ethnicity: white(36.2%), black (53.6%), Hispanic/Latina(12.2%), Native American(1.2%), and other(1.2%). Age: 18–34 (30%) 35–55 (64%), 56 ≤ (6%)	gender-specific and culturally informed services that were provided by peer mentors for example assisting clients with finding transportation, learning bus schedules, or practicing for a driver’s license test; making and attending medical and behavioral health appointments,..	Psychosocial functioning -days of drug use - depression, anxiety, and hallucinations - the rate of employment, attendance at treatment related appointments	-significant reductions in days of drug use, depression, anxiety, and hallucinations -higher rates of employment, attendance at treatment-related appointments, and job training programs at follow-up - 55% of the program participants were placed in housing - Four principles that guide the effective provision of services to these women include: (1) the use of peer support, (2) provision of flexible resources in alow demand environment, (3) supportive program leadership, and (4) treatment delivered for and by women.
Kelly & Pich [20]	Community-based PTSD Treatment for Ethnically Diverse Women Who Experienced Intimate Partner Violence: A Feasibility Study	USA	pretest/post-test intervention	-Gender (Women) -Immigrant -Race/ ethnicity -IPV -PTSD	22	Age: 36.7 (Range:19–58) Education (years): 10.7 (0–14) Years in USA: 17.9 (7–40) Time with abuser (years): 6.2 (1–17) Separated from abuser (months): 9.3 (2–24) Immigration status Group 1: Spanish-speaking immigrants: Legal resident: 3 Undocumented: 7 Groups 2–3: English proficiency: Spanish-speaking group US citizen: 12 None to minimal: 6 Moderate to fluent: 4	-The initial intervention consisted of 6–10 sessions of weekly psychotherapy groups, using a synthesis of supportive psychotherapy,including psycho-education and self-care strategies. -The initial intervention was revised for the third group, with the addition of ACT.	-PTSD symptoms -Depression symptoms -HrQOL -Self rated health -Self efficacy -Social support	Compared to the baseline, PTSD symptoms had decreased at 6 months post-intervention (*p =* 0.003); depression symptoms decreased at all post-intervention time-points (2 weeks (*p* ≤ 0.002), 3 months (*p* ≤ 0.004), and 6 months post- intervention (*p* ≤ 0.000)); Self-reported mental health-related QoL improved at 3 months (*p =* 0.013) and 6 months post intervention (*p* = 0.003); and Self-efficacy significantly improved at 6-month follow-up (*p* = 0.020). Improvement of physical health-related QoL and perceived social support scores at each time-point compared with baseline were not statistically significant.
Montgomery [21]	Adapting a Brief Evidence-Based Intervention for Text Message Delivery to Young Adult Black Women	USA	Phase 2: RCT	-Gender (women) -Age (young adult) -Ethnicity (black)	Baseline Surveys Submitted (*n* = 92); Randomized to the Intervention Group(*n* = 45) & control group(*n* = 47)	Age: 21.07 ± 1.73 (Range: 18–24) Ethnicity: Black Gender: Female Education: Some college, but no bachelor degree (59.1%) Employment: Employed, working 1–39 h per week (65.9%)	sending 24 text message (Intervention Group: regarding sexual health; Control group: regarding diet and exercise)	-Primary outcome: Condom use -Secondary Outcomes: • condom use self-efficacy • condom use intentions • sexual relationship power	-Between baseline and follow-up, condom use frequency increased among participants in both study groups. However, there was no significant time by group interaction. Furthermore, while condom use self-efficacy and intention significantly increased among participants in both groups, no time by group interaction was found. Intention was identified as a main predictor of condom use at baseline and follow-up.
Kisler [22]	Minority Stress and HIV Risk Behavior among HIV-Positive Bisexual Black Men with Histories of Childhood Sexual Abuse	USA	RCT	-Gender(Men) -Disease (HIV-positive) -Ethnicity (Black) -Sexual Orientation (Men sex with men and women)	Baseline (*n* = 117); The ES-HIM intervention condition / the HP control condition sample included 88 (44 per condition)	Age: 45.77 ± 8.81 (Range:24–67) Ethnicity: Black Gender: male Education: High school diploma (35.9%) Employment: Unable to work or disabled (55.2%) Monthly Income: $833–$1042 (38.9%) Marital Status: Never married (75%)	Providing group discussions (intervention condition: topics related to reducing HIV risk behavior and symptoms of depression and posttraumatic stress; Control condition: topics related to general health and medication adherence)	- HIV risk behavior (Main outcome) -Perceived Internalized racism -Perceived internalized homophobia	-Internalized racism did not decrease over the four time points for either the group as a whole, or by intervention condition. -Internalized homophobia, significantly diminished over the four time points for the group as a whole, but no differences between the ES-HIM intervention and Health Promotion control condition were found. -Finally, frequency of HIV risk behavior (i.e., intercourse without a condom) also decreased from baseline to post-intervention assessment for the group as a whole, but no intervention effects were found.

*Abbreviations: PTSD* Post Traumatic Stress Disorder, *IPV* Intimate Partner Violence, *ACT* Acceptance and Commitment Therapy, *HrQoL* Health-related Quality of Life, *RCT* Randomized Controlled Trial, *ES-HIM* the Enhanced Sexual Health Intervention for Men, *HP* the Health Promotion

Social identities (e.g. gender, race/ethnicity, age, migration, sexual orientation, & homelessness) were taken into account in the selected studies. Moreover, participants experienced complex life circumstances (such as PTSD, IPV, history of childhood sexual abuse, substance use or co-morbid substance use, and mental illness) that could interfere with the health outcomes. In this regard, the studies attempted to consider the intersection of these co-occurring social identities and conditions through the intersectionality framework in order to achieve a more comprehensive understanding of the issue. The intersection of gender and race/ethnicity had been addressed in three of the selected studies [20–22]. However, the other social identity variables were only addressed separately in each of these studies e.g. the status of migration and its subgroups [20], age [21], sexual orientation, HIV, and the history of childhood sexual abuse [22], as well as homelessness, substance use or co-morbid substance use, and mental illness [18]. Table 3 shows the intersections of the different variables which were addressed in the selected studies.

**Table 3:** Intersecting categories in included articles (*n* = 4)

Categories	IPV and PTSD	Substance use	Histories of Childhood Sexual Abuse	HIV	Sexual orientation	Homeless-ness	Age	Migration	Race/ethnicity	Gender
**Gender**	1	1	1	1	1	1	1	1	3	X
**Race/ethnicity**	1	0	1	1	1	0	1	1	X	
**Migration**	1	0	0	0	0	0	0	X		
**Age**	0	0	0	0	0	0	X			
**Homelessness**	0	1	0	0	0	X				
**Sexual orientation**	0	0	1	1	X					
**HIV**	0	0	1	X						
**Histories of Childhood Sexual Abuse**	0	0	X							
**Substance Use**	0	X								
**IPV and PTSD**	X									

The results of the synthesis of the application of intersectionality principles are presented in Table 4.

**Table 4:** Synthesis of application of intersectionality in included studies

	Possible response to each question	Intersecting Categories	Multilevel Analysis	Power	Reflexivity	Time & Space	Diverse Knowledge	Social Justice & Equity
**Problem Identification**	Yes	11 (91.66%)	6 (75%)	8 (66.66%)	0	1 (25%)	5 (62.5%)	3 (37.5%)
	No	1 (8.33%)	2 (25%)	4 (33.33%)	0	3 (75%)	3 (37.5%)	3 (37.5%)
	Unclear	0	0	0	4 (100%)	0	0	2 (25%)
	Number of questions ^a^	12	8	12	4	4	8	8
**Design & implementation**	Yes	8 (100%)	1 (25%)	19 (79.16%)	1 (25%)	3 (75%)	4 (50%)	4 (50%)
	No	0	3 (75%)	1 (4.16%)	0	0	0	0
	Unclear	0	0	4 (16.66%)	3 (75%)	1 (25%)	4 (50%)	4 (50%)
	Number of questions ^a^	8	4	24	4	4	8	8
**Evaluation**	Yes	0	1 (25%)	5 (62.5%)	0	Not relevant ^b^	4 (50%)	0
	No	4 (100%)	3 (75%)	3 (37.5%)	0	Not relevant ^b^	4 (50%)	4 (50%)
	Unclear	0	0	0	4 (100%)	Not relevant ^b^	0	4 (50%)
	Number of questions ^a^	4	4	8	4	0	8	8
**Overall**	Yes	19 (79.16%)	8 (50%)	32 (72.72%)	1 (8.33%)	4 (50%)	13 (54.16%)	7 (29.16%)
	No	5 (20.83%)	8 (50%)	8 (18.18%)	0	3 (37.5%)	7 (29.16%)	7 (29.16%)
	Unclear	0	0	4 (9.09%)	11 (91.66%)	1 (12.5%)	4 (16.66%)	10 (41.66%)
	Number of questions ^a^	24	16	44	12	8	24	24

The numbers in each cell are the sum and percentage of “yes”, “no” or “unclear” responses to questions on each stage of “problem identification”, “design & implementation”, and “evaluation”

^a^Total number of questions of each principle for all included studies. For example, the number of questions of *“Intersecting Categories”* principle in stage of “problem identification” (*n* = 3), totally is 12 for four included studies

^b^The checklist had no any question for the principle of *“time and place”* in the stage of “evaluation”

Overall, the principles of intersectionality were mainly addressed in the stages of “problem identification” as well as “design & implementation”. On the other hand, the principles were either difficult to observe or judge in the “evaluation” stage. Based on the findings, the reviewers responded *‘yes’* to 79.16% of the questions regarding “*intersecting categories”* and 72.72% of the questions regarding “*power”*. In other words, the application of these two principles is regarded considerable, particularly in the stages of “problem identification” as well as “design & implementation” of the interventions. The studies were focused on vulnerable groups. Furthermore, the social, economic, and cultural circumstances as well as the needs of the target populations had also been emphasized at several stages of the interventions. However, the impact of power structures – such as policies – had received less attention in creating or responding to the problem. “*multilevel analysis*” was recognized as the principle with less attention in the selected studies. Overall, half of its items have not been observed, and based on the results, this principle garnered less attention in the stages of “design & implementation” and “evaluation”. Despite having addressed the intersection of different factors at the individual, organizational, and/or policy levels during the stage of problem identification, most studies have conducted the interventions at micro levels (personal & interpersonal relationships) and did not aim at bringing about changes at the structural levels. Although David et al. [19] claimed that they have focused on describing a systems level intervention, their findings can contribute to individual therapists who are dealing with women suffering from multiple interlocked problems including homelessness, substance use, behavioral health disorders, and trauma histories. In term of “*time & space*” principle, the selected studies addressed the process of problem framing over time and across different locations as well as the possible changes in the intersection of the social variables and the experiences of inequity.

Kelly [23] was the only researcher to mention the political atmosphere in the first decade of the twenty-first century in the US as well as the Latinas/os’ experiences of discriminative political and legal circumstances. The selected studies used a variety of quantitative and qualitative evidence in order to identify the problems and design or evaluate the interventions; in other words, these studies have only relatively focused on the principle of *‘"diverse knowledge*". Nevertheless, the target group’s perspective (which is an important source of knowledge in intersectionality) was only taken into account in two studies. David et al. [19] evaluated their project by conducting interviews and focus group discussions in order to collect the viewpoints of the clients, peer mentors, and the project staff about the probable barriers and facilitators of the implementation of the program. Kelly and Pich [20] reported that the need to conduct the study had been identified and advocated for by the community partner as well; additionally, these researchers performed interviews and focus group discussions with the staff and participants to assess the acceptability and feasibility of the intervention. Based on these qualitative data, they decided to adopt the intervention and revised the study design accordingly. Moreover, the selected studies have examined vulnerable groups with respect to the principle of “*social justice & equity”*; thus, it can be argued that the interventions were partly conducted with the aim of reducing the experiences of inequities. However, none of the included studies had considered the reduction of inequity as a criterion of success. In addition, they did not even evaluate the idea of ensuring that the interventions may not lead to further inequalities in some populations. The overall results of the application of the two principles of *‘"reflexivity"* and *"social justice & equity"’* also indicated that the participant responded *‘unclear’* to a considerable number of the questions. It means that it is difficult to make an accurate judgment about these principles based on the writing styles of the previous studies.

Moreover, only one study reported the effectiveness of the intervention. Kelly and Pich [20] conducted a supportive psychotherapeutic pilot intervention using a Community-Based Participatory Research (CBPR) approach. It was performed on Latinas with PTSD who have experienced IPV. This intervention was feasible and acceptable because of the flexibility and the modifications to respond to the community partners’ needs. Such an intervention, however, was a pretest-posttest intervention without a control group, and the researchers asserted that it caused difficulty to control the effects of the intervention throughout the time or the effects of social interactions as a part of the intervention on mental health. The small sample size and the inconsistent doses of the interventions on a varying number of attendances at group sessions were regarded as the other limitations of the present study. The effectiveness of the interventions was not supported in the two studies by Montgomery [21] and Kisler [22]. Finally, David et al. [19] reported significant improvement in the outcomes of psychosocial functioning; on the other hand, because of the observational nature of their study, it was not possible to judge the interventions in terms of effectiveness.

## Discussion

Responding to multidimensional and complex issues such as health inequalities among marginalized and oppressed populations requires multifaceted and new multidimensional approaches [11]. We examined the application of intersectionality in designing and implementing health interventions according to the stages heuristic model (problem identification, design & implementation, and evaluation). This review study can help inform the researchers and policymakers about the use of intersectionality as an innovative approach in order to solve these complicated problems and show the probable gaps in this area of research.

According to the results, the studies which were included in the review process were conducted between 2013 and 2016; they were limited to the studies which were conducted in the United States and focused on using the intersectional approach in the stages of “problem identification” and “design & implementation”. It also shows that the application of intersectionality in interventions and programs is a relatively new topic in the field of health and therefore needs to be further addressed by the policymakers and researchers so as to help reduce health inequalities. Similarly, Couto et al. [13] performed a literature review and concluded that despite the potential of intersectionality in supporting empirical studies and formulating the health policies to reduce inequalities, there is still a need for new measures to move towards the incorporation of intersectionality in the field of health.

The present study indicated that *“intersecting categories”* and *“power”* have received greater attention in the selected studies. Focusing on the multiple intersections of social identities –as the most basic tenet of intersectionality [11] –has helped identify the target groups accurately. Intersectionality highlights the differences between and within the groups, with a focus on vulnerable populations. These findings suggest that the intersectionality approach can help health researchers and policymakers accurately identify the most vulnerable groups and hear their voices. Based on our findings, gender was considered as the focus of attention in all the studies; in addition, the most frequent intersection was observed between the variables of gender and race/ethnicity, which was addressed in three of the studies [20–22]. In this regard, Larson’s review study [8] also confirmed that the focus has been on gender across all the selected empirical articles.Moreover, a great number of empirical studies conducted in HICs highlighted how gender could intersect with race/ethnicity/caste. These findings can be justified by the fact that intersectionality is rooted in Black feminist scholarship in the exclusion of Black women from White feminist discourse and antiracist discourse in the 1990s. Based on the intersectionality approach, single or dual analytical categories such as race and gender can offer limited explanatory power [11]. Thus, it is suggested that future research should pay more attention to multiple dimensions of social identities in order to improve the analysis of health inequalities.

Regarding the principle of *“diverse knowledge*”, although the selected studies used different qualitative and quantitative evidence to explain the problem identification, design, and implementation of the interventions, the target group’s perspective was only investigated in the two studies by David et al. [19] as well as Kelly and Pich [20]. The relationship between power and knowledge production is an important issue in the intersectionality perspective. The pressure of power that runs through the process of knowledge production can be reduced during the health planning and/or policymaking by including of the viewpoints of marginalized individuals and groups who are excluded from the knowledge production [10]. Moreover, the interventions mainly focus on systems and power structures such as healthcare systems, public policy, laws, economy, and education rather than the individuals or groups [23]. It is also noteworthy that most of the studies have conducted the interventions at the micro level (personal and interpersonal relationship), and have not aimed at bringing about any changes at the structural levels. Therefore, future research should pay more attention to power structures at macro levels in order to reach the power balance and establish social justice.

Intersectionality can also address power at micro and macro levels of society through reflexivity [10]. The “*reflexivity”* principle helps correctly identify the problems and directs the health researchers’ or policy makers’ minds toward the acceptance and involvement of stakeholders, particularly the marginalized groups, at various stages of planning. Furthermore, the changes in time and space might have an impact on the nature of determinants of health outcomes. Besides, focusing on the intersections of these factors can be helpful in developing an accurate identification of the problems and designing the necessary interventions. However, the findings suggest that the researchers have paid less attention to the principles of *“reflexivity”* as well as *“time and space”*. It is probably because the articles have not been written in such a manner that would allow accurate judgment regarding these items, despite the fact that social justice is regarded as an important principle of intersectionality. On the other hand, none of the selected studies had considered the reduction of inequity as a criterion of success; they had not also assured that the health intervention or program may cause further inequities among other groups. Therefore, we cannot discuss the impact of interventions on inequity. It seems that it is useful to inform the researchers about the principles of intersectionality in eliminating the shortcomings of intersectionality application.

Overall, there was no evidence regarding the effectiveness of such interventions in most of the selected studies. It can also be interpreted that there is not enough evidence for the effectiveness of interventions with an intersectionality approach. Thus, we suggest conducting further research with a pragmatic approach in producing evidence for the effectiveness of such interventions provided that the principles of intersectionality are not fully addressed in the existing studies. The results of the review of the included studies indicated the lack of adherence of researchers to the principles of intersectionality or the lack of compliance with these principles, particularly “*reflexivity”* and *“social justice and equity”.* Hence, preparing standard guidelines for the implementation of intersectionality in health planning would be necessary. In this regard, several researchers have emphasized the need for new types of expertise regarding intersectional perspective and focusing on all the principles of intersectionality [24, 25]. The Intersectionality-based Policy Analysis Framework is recognized as one of the practical instruments in this area, which is designed to evaluate the effects of policy on individuals and their experiences of health inequalities [12]. The proposed checklist in this study was developed based on the results of the literature review and the existing frameworks; it was also arranged according to the stages heuristic model including several intersectionality principles. Hence, it can be useful in this respect.

### Strengths and limitation

To the best of our knowledge, this is the first scoping review study that aimed to examine the extent of the application of the intersectionality in designing and implementing the health interventions and programs. The researchers in the present study used a checklist which focuses on different intersectionality principles at stages heuristic model. Accordingly, the reviewers were able to objectively extract the data on the application of intersectionality. Since there are limited approaches for the application of the intersectionality in health system contexts, such approaches have only been employed by a few researchers [24]. Therefore, the proposed checklist can be considered as a helpful instrument to apply intersectionality and reduce the probable gaps between theory and practice in this field.

The researchers attempted to make the search strategy as comprehensive as possible and they used a wide range of keywords as well. Nevertheless, only a few studies were eventually included in our review study. A stronger focus on the databases as search resources and the lack of review of gray literature due to our limitations during the research process might be regarded as the limitations of the present study. Moreover, intersectionality is a relatively new concept in the field of health and its terminology has not been standardized yet. According to our inclusion criteria, the studies had to clearly state the application of “*intersectionality*” in designing and implementing the intervention or program in order to be selected by the researchers; thus, it is possible that some studies have been conducted to reduce inequalities and have taken into account the principles of intersectionality in designing and implementing the health interventions and programs, but they might not have directly mentioned intersectionality. However, this limitation might be resolved at the search stage if the objective tools are employed at the screening stage in future studies.

## Conclusion

This review study can inform the target audiences about the application of the intersectionality in designing and implementing health interventions and programs; it can also highlight the existing gaps in this area. Given the small number of included studies, there is still insufficient evidence within empirical studies to accurately show the implication of intersectionality in designing and conducting the health interventions and programs. There is a need to address the principles at various stages of health policies and interventions in order to operationalize intersectionality. To this end, the development and availability of user-friendly instruments may help researchers and health policymakers apply intersectionality appropriately.

## Supplementary Information

**Additional file 1.** Search syntax in PubMed.

## Data Availability

All the data which were generated or analyzed during this study are included in this article.

## Abbreviations

LMICs: Low- and Middle- Income Countries
HICs: High Income Countries
PRISMA: The Preferred Reporting Items for Systematic Reviews and Meta-Analyses
PTSD: Post Traumatic Stress Disorder
IPV: Intimate Partner Violence
ACT: Acceptance and Commitment Therapy
HrQoL: Health-related Quality of Life
RCT: Randomized Controlled Trial
ES-HIM: Enhanced Sexual Health Intervention for Men
HP: Health Promotion
CBPR: Community Based Participatory Research
