# Supplementary material for: “Applying Intersectionality in designing and implementing health interventions: a scoping review”
Source: BMC Public Health. 2021 Jul 16;21:1407. doi: 10.1186/s12889-021-11449-6 (PMC8283959; doi:10.1186/s12889-021-11449-6)
Supplement: Supplementary file 1 — Additional file 1. Search syntax in PubMed. [file 12889_2021_11449_MOESM1_ESM.docx]

| **Additional file 1- Search syntax in PubMed** |
| --- |
| (Intersectionality OR “Intersectionality Health” OR “intersectionality approach” OR “intersectionality framework” OR “intersectionality perspective” OR “intersectionality informed” OR “intersectionality lens” OR “intersectionality based” OR “intersectionality theory” OR “intersectionality research” OR “Intersectional policy” OR “intersectional approach” OR “Intersectional analysis” OR “intersectional paradigm” OR “intersectional lens” OR “Intersectional method” OR “Intersectional framework” OR “Intersectional perspective” OR “Intersectional strategies”) AND (Intervention OR “government Program” OR (Program AND Government) OR “Government Sponsored Program” OR (Program AND “Government Sponsored”) OR “Government-Sponsored Program” OR (Program AND “Government-Sponsored”) OR “Health policy” OR (Policy AND Health) OR “National Health Policy” OR (“Health Policy” AND National) OR (Policy AND “National Health”) OR “Health Planning” OR (Planning AND Health) OR (Planning AND Health and Welfare) OR (Health and “Welfare Planning”) OR “Health Care Reform” OR (Reform AND “Health Care”) OR “Healthcare Reform” OR (Reform AND Healthcare) OR “Accountable Care Organization” OR (“Care Organization” AND Accountable) OR (Organization AND “Accountable Care”) OR “Delivery of Health Care” OR “Healthcare Delivery” OR (Delivery AND Healthcare) OR “Delivery of Healthcare” OR “Health Care Delivery” OR (Delivery AND “Health Care”) OR “Health Care System” OR (System AND “Health Care”) OR “Healthcare System” OR (System AND Healthcare) OR “Community-Based Distribution” OR “Community Based Distribution” OR (Distribution AND Community-Based) OR “Distributional Activity” OR (Activity AND Distributional) OR “Health Service” OR (Service AND Health) OR “Health Plan Implementations” OR (Implementation AND “Health Plan”) OR (“Plan Implementation” AND Health) OR (“Plan Implementations” AND Health) OR (Guideline AND “Health Planning”) OR (Guidelines AND “Health Planning”) OR “Health Planning Guideline” OR (“Planning Guideline” AND Health) OR “Guidelines for Health Planning” OR “Health Planning Recommendation” OR (“Planning Recommendation” AND Health) OR (Recommendation AND “Health Planning”) OR “Health Services Evaluation” OR (Evaluation AND “Health Services”) OR “Healthcare Research” OR (Research AND “Health Services”) OR (Research AND “Medical Care”) OR “Health Care Research” OR (Research AND “Health Care”) OR “Medical Care Research” OR “Action Research” OR (Research AND Action) OR “Health Services Research” OR (“Health Program” AND National) OR “National Health Program” OR (Program AND “National Health”) OR “National Health Insurance” OR (“Health Insurance” AND National) OR (Insurance AND “National Health”) OR (“Health Service” AND National) OR “National Health Service” OR (Service AND “National Health”) OR (Organization AND “Health Planning”) OR “Health Planning Organization” OR (“Planning Organization” AND Health) OR (“Control Policy” AND Social) OR (Policy AND “Social Control”) OR “Social Control Policy” OR (“Social Control” AND Formal) OR “Formal Social Control” OR “Social Control” OR (Control AND Social) OR Regulation OR “Facility Regulation and Control” OR (Regulation AND Facility) OR “Facility Regulation” OR “Facility Control” OR (Control AND Facility) OR “Government Regulation” OR (Regulation AND Government) OR “Government Regulation and Oversight” OR “Policy making” OR (Making AND Policy) OR “Policy Development” OR (Development AND Policy) OR “Policy Analysis” OR (Analysis AND Policy) OR “Administration and Organization” OR “Administrative Technique” OR (Technique AND Administrative) OR “Administrative Technic” OR (Technic AND Administrative) OR Logistics OR Supervision OR Administration OR (Coordination AND Administrative) OR “Administrative Coordination” OR “Public Health Systems Research” OR “Availability of Health Services” OR “Health Services Availability” OR (Accessibility AND “Health Services”) OR “Access to Health Care” OR “Accessibility of Health Services” OR “Health Services Geographic Accessibility” OR “Program Accessibility” OR “Accessibility AND Program” OR “healthcare Quality Assurance” OR (Assurance AND “Healthcare Quality”) OR (“Quality Assurance” AND Healthcare) OR “Health Care Quality Assurance” OR “Healthcare Quality Assessment” OR (Assessment AND “Healthcare Quality”) OR (“Quality Assessment” AND Healthcare) OR (Quality Assessment AND Health Care) OR “Health Care Quality Assessment” OR “Managed Care Program” OR (Program AND “Managed Care”) OR “Managed Health Care Insurance Plans” OR “Managed Care” OR (Care AND Managed) OR “Insurance Case Management” OR (“Case Management” AND Insurance) OR (Management AND “Insurance Case”)) |
